# Predictors of long-term resistance exercise adherence: evidence from a large cohort of mobile app users of various experience levels

**DOI:** 10.3389/fspor.2026.1855668

**Published:** 2026-08-10

**Authors:** Federica Conti, Thiago Marzagão, Andrew J. Galpin, Brad J. Schoenfeld

**Affiliations:** 1 Human Performance Center, Parker University, Dallas, TX, United States; 2 Fitbod Inc., San Francisco, CA, United States; 3 Department of Exercise Science and Recreation, Lehman College, City University of New York, New York, NY, United States

**Keywords:** behavior change, digital health, exercise adherence, lifestyle and behavior, physical activity

## Abstract

**Background:**

Digital fitness applications offer unprecedented access to structured training programs, yet the behavioral factors that predict sustained engagement in real-world settings remain incompletely understood.

**Methods:**

This observational study analyzed data from 389,481 adult digital fitness app users (mean age: 34.7 ± 9.9) of various experience levels followed for twelve months from their first recorded workout. Long-term adherence was defined as completing at least one workout per week, allowing up to six missed weeks. Adherence trajectories were examined, along with associations between early training behaviors (training frequency, workout duration, exercise composition, equipment diversity), demographic factors, and time to dropout. Effect modifications by sex, workout duration, and training experience were also investigated.

**Results:**

Adherence declined steadily over time, with 10.1% of beginner users remaining adherent at 12 months (i.e., 52 weeks). The median dropout time was 19 weeks. Higher sustained participation was observed in older vs. younger (51+: 13.3%; 18–40: 8.4%), male vs. female (M: 11.4%; F: 7.9%), and more experienced users (intermediate: 18.3%; advanced: 25.8%). Training consistency during the first 28 days was the strongest predictor of adherence and exhibited a protective association that attenuated over time. Greater diversity in equipment use and higher emphasis on resistance exercise were also associated with lower dropout risk. Longer workout duration was associated with improved adherence among users who trained more frequently, particularly early in follow-up.

**Conclusions:**

Early consistency and structured training behaviors were strongly associated with long-term app-recorded exercise adherence among beginners, with relatively modest differences based on age and sex. These findings suggest that frequent training sessions and engagement with resistance-based exercise during the initial stages of exercise adoption may be relevant behavioral correlates of sustained engagement.

## 1 Introduction

Regular exercise is one of the most effective strategies for promoting and maintaining physical and mental health across the lifespan (1–3). In recent years, digital technology has progressively transformed how people shape, structure, and monitor their fitness goals. Thanks to the proliferation of mobile applications, wearable devices, and online training platforms, personalized programming and performance feedback are now readily accessible. These commercial tools are conceived to reduce the need for in-person guidance, offer flexible workout schedules, and lower barriers to entry by providing clear, structured, and individualized exercise routines (4, 5).

Nevertheless, the degree to which such platforms improve long-term exercise adherence—particularly among beginners—remains insufficiently understood (6). Even with unprecedented access to equipment, instructional content, and tailored programs, many individuals still struggle to initiate and sustain physical activity behaviors that yield meaningful health benefits (7). Global health authorities recommend at least 150–300 min of moderate-to-vigorous activity per week, including both aerobic and resistance-based exercise (8), yet adherence to these guidelines remains low across many countries. In 2022, only about 22.5% of US adults aged 25 years and older were found to engage in regular physical activity as per national recommendations (9), with even lower rates (13.9%) observed among adults over the age of 65 (10). This disconnect suggests that access alone does not guarantee behavior change (11) and understanding how to encourage the establishment and maintenance of sound exercise habits in the general population remains a critical public health priority.

Multiple categories of factors may be implicated in long-term exercise adherence (12). Demographic and individual characteristics such as age, sex, socioeconomic status, and baseline fitness level may influence motivation, physical capability, and preferred training modalities (13, 37). Exercise beginners may further be exposed to higher dropout risk due to psychological barriers such as lack of self-confidence or self-efficacy, and fear of external judgement (14, 15). Workout patterns that facilitate the formation of stable exercise habits by encouraging greater training frequency and consistency may therefore be beneficial in these early stages. Exercising at regular intervals gradually strengthens automaticity and reduces reliance on conscious motivation (16), while the successful completion of progressively challenging exercise sessions may enhance self-efficacy through mastery experiences, thus reinforcing individuals' confidence in their ability to sustain regular participation. Last but not least, exercise experiences perceived as enjoyable and rewarding may promote intrinsic motivation, thereby increasing the likelihood of long-term adherence. By this view, striking the right balance between challenge and attainability in the first few weeks of training through the appropriate combination of volume, intensity and variation is paramount to promote skill development and minimize injury risk, ultimately enhancing overall enjoyment and exercise adherence. Environmental and contextual cues such as adequate access to fitness equipment and facilities (17), continuous social support, and personal accountability (18), may further help shape health-promoting physical activity behaviors to an appreciable extent.

To date, much of the scientific research on exercise adherence has focused primarily on specific populations (e.g., clinical groups, older adults) or structured interventions (e.g., supervised programs), with limited applicability to real-life scenarios. Moreover, clinical studies on physical activity behaviors mostly rely on self-reported measures, notoriously prone to recall bias and poor accuracy (19). Critically, when not exposed to structured training stimuli and targeted behavioral support, beginners may be prone to early disengagement from regular exercise, yet comparatively little research has specifically addressed adherence outcomes in this selected cohort. To this scope, by capturing real-world physical activity patterns across various experience levels, large-scale data from fitness apps can help identify significant predictors of adherence based on objective records of user behavior—including frequency, volume, progression, and exercise selection—over long timescales.

The present observational study combines app-based tracking and survival modelling to investigate the association between demographic variables, early exercise patterns, and sustained app-recorded physical activity engagement in a large sample of fitness app users of various experience levels. Within this framework, we hypothesized that: (i) higher early training frequency would predict longer adherence; (ii) greater diversity of exercise types or modality (i.e., strength, cardio, mobility) would extend survival; (iii) shorter workouts could be protective if they promote sustainability, as shorter exercise sessions may facilitate initial adherence by lowering perceived time demands and reducing barriers to participation; (iv) dropout risk would be lower in male compared to female users. The findings from this study will contribute to the broader understanding of how individuals successfully transition from exercise initiation to long-term adherence and will provide data-driven and ecologically valid insights that can inform program design, beginner onboarding strategies, and tailored interventions to improve exercise consistency in real-world settings.

## 2 Methods

### 2.1 Preregistration and study framework

This study forms part of a preregistered project on the Open Science Framework (OSF) titled “*Determining patterns of exercise selection, training behaviors, and fitness outcomes in real-world settings from fitness app data”* (https://ofs.io/gufy9/overview). The present analysis corresponds to the preregistered component focused on predictors of long-term adherence in exercise beginners using survival analytic methods.

### 2.2 Human research ethics

Sensitive data collected for the present study included users' age only. Users were assigned a 32-digit alpha-numeric identifier, and their demographic details and individual data were stored exclusively against this number. All statistical analyses were conducted on de-identified data. Therefore, the Institutional Review Board at Parker University confirmed that this work did not constitute human subjects research and ethical approval was not necessary for the conduct of this study (protocol number: PUIRB-2025-33).

### 2.3 Data source and user sample definition

Data for this observational study were derived from the commercially available smartphone fitness application developed by Fitbod Inc., providing individualized workout recommendations based on user goals, experience level, and training preferences, including primarily strength-based activities, but also cardio and mobility routines. As such, users may freely initiate, modify, postpone, or skip recommended workouts according to their preferences. The app records detailed information about each session, including timestamped workout logs, exercise selections and modifications, sets, repetitions, loads, workout duration, and general workout type or composition (i.e., strength, cardio, mobility). These data are entered by users via the smartphone interface and automatically stored on Fitbod's secure servers. By connecting the Fitbod fitness app to their smartwatches or smartphones, users can further import external workout sessions recorded using third party companies, such as Strava, Fitbit, and Apple Health/Android Connect. For the present study, a formal data-sharing agreement between the research team and Fitbod Inc. allowed access to a fully anonymized dataset comprising user workout histories from the designated observation period. All personal identifiers were removed prior to data transfer and researchers did not have access to user identity or account details.

The final dataset was extrapolated from recorded workouts performed between January 18th, 2016, and May 11th, 2025. This time window was selected to maximize data pooling while also ensuring data consistency and comparability despite changes and updates to the app user interface and onboarding procedure. Eligible users were selected based on the following predefined inclusion criteria:

1. Age ≥ 18 years at the time of account creation.
2. Sex (Male/Female) specified.
3. Self-identified as beginners (<1 year of experience), intermediate (1─4 years), or advanced (4+ years) during the app onboarding process.
4. Completed at least one workout session per week during the initial 28-day period following their initial workout.
5. Fully observable through day 365 (i.e., data logged and successfully recorded for 12 months, i.e., 52 weeks) following their initial workout.

The specific choice of the one-workout-per-week frequency threshold in the initial 28-day period was motivated by the attempt to maximize data retention while simultaneously capturing early trends towards weekly training consistency. This inclusion criterion necessarily excluded users who disengaged immediately following app onboarding and therefore provided insufficient exercise exposure to meaningfully characterize early training behaviors. Accordingly, the present analyses were designed to identify predictors of sustained app-recorded exercise engagement among users who demonstrated initial participation, rather than predictors of exercise adoption or immediate dropout across the broader population of new app users.

The above-described data selection, cleaning and preprocessing steps are summarized in Supplementary Figure S1.

### 2.4 Measures

The primary outcome variable of the present observational study was long-term exercise adherence within the beginner cohort, defined as having completed at least one workout session per week between days 29 and 365, regardless of workout source, i.e., Fitbod vs. externally imported, and allowing up to six exceptions to account for likely real-life scenarios preventing exercise participation such as holidays, sick days, and other unforeseeable circumstances. This allowance was incorporated to acknowledge that temporary interruptions are common and do not necessarily represent abandonment of an exercise routine. Our objective was therefore to identify users who maintained sustained exercise engagement over the long term rather than those who achieved uninterrupted weekly participation. Based on this definition, app engagement was therefore treated as a proxy for exercise adherence. The twelve-month (i.e., 365 days) observational period was chosen to ensure reasonable data retention for statistical analyses and was further informed by prior evidence of significantly reduced long-term adherence in unsupervised fitness settings at 12 months, i.e., 52 weeks [e.g., <4% of 5,240 new gym members (20)], thus suggesting that longer follow-up periods would compromise statistical power.

Exercise-related predictor variables were operationalized using data extracted from each user's first 28 days following their initial recorded workout on the app. Several domains were quantified to capture differences in training frequency, workout composition, equipment use, and subscription patterns. Training frequency was assessed using both the total number of workouts completed in the first 28 days (including both app-generated and externally imported sessions) and the number of app-generated workouts specifically. For both variables, we calculated the corresponding total time spent exercising over the initial 28-day period. To characterize workout composition, we computed the percentage of completed workouts that included at least one mobility or cardio exercise, and the percentage of all exercises performed that were classified as strength based. Training frequency and consistency were captured through the number of distinct days over the initial 28-day period on which users logged at least one workout of any type (either app-generated or external), as well as the number of days within this initial window that included strength, cardio, or mobility components. During onboarding, users were asked to indicate which types of exercise equipment they had available. This information was then used to derive the number of distinct equipment types that users had at their disposal to complete their sessions, potentially reflecting the variety of exercises included in their workout routines. Finally, subscription-related variables were also considered, namely whether the user purchased a paid app subscription in the 28 days following their initial workout.

### 2.5 Statistical analyses

Statistical analyses were performed using R (version 4.5.2; R Foundation for Statistical Computing) and Python (version 3.14.0; Python Software Foundation). Descriptive statistics were examined to characterize the user sample based on self-reported experience level. User demographic characteristics were summarized by computing the proportion of males and females within each group and by examining the relative age distributions across standardized age groups (e.g., 18–30, 31–40, 41–50, etc.), reported in terms of both the number of users and corresponding percentages within each sample.

For the beginner cohort, early exercise behaviors during the initial 28-day period were summarized via the distributions of all predictor variables, including indicators of training frequency and consistency, workout duration, exercise-type composition, and preferred training modality. Total and app-generated workout durations were highly right-skewed due to occasional implausibly long sessions (e.g., max cumulative exercise duration over 28 days = ∼45,819 h), which were considered highly unlikely to represent true exercise behavior and instead most likely reflected logging artifacts, such as sessions inadvertently left running, duplicated records, or synchronization errors. To reduce the influence of these extreme values, duration variables were winsorized at the 98th percentile prior to analysis. This threshold was carefully selected as a pragmatic compromise to retain meaningful variation in workout duration while limiting undue influence from unrealistic outliers (winsorized max cumulative exercise duration over 28 days = ∼71 h). Accordingly, winsorization was applied consistently across descriptive and inferential analyses to ensure robustness of duration-related estimates. Importantly, because the above artifacts appeared to affect duration exclusively rather than user participation itself, we elected to winsorize rather than exclude affected observations entirely thereby preserving otherwise valid information on workout frequency, exercise composition, and adherence.

### 2.6 Survival analysis

To examine patterns of long-term exercise adherence, survival analyses were conducted using time-to-event methods. For each user in the beginner cohort, follow-up began on day 29 after their first recorded workout. The primary event of interest was loss of adherence, also referred to as dropout, defined as the accumulation of more than six weeks with no active days during the observation window spanning days 29 to 365 (i.e., weeks 5 to 52). Based on this definition, dropouts could not occur before week 11. Accordingly, users were considered at risk for dropout beginning in week 11, and those who remained adherent throughout the entire follow-up window were treated as right censored at week 52. A survival object was therefore constructed using the observed time to dropout for non-adherent users and the censored time of 52 weeks for adherent users.

Adherence trajectories were estimated using Kaplan–Meier (KM) survival curves. Such curves were generated both for the overall beginner sample and separately based on demographic variables of interest, specifically sex (male/female) and age group. Age categories (18–30, 31–40, 41–50, and ≥51 years) were selected *a priori* to ensure adequate sample size within each stratum and to reflect meaningful differences in exercise behavior across the lifespan. Equality of survival functions across groups was assessed using log-rank tests. For all KM displays, the number of users at risk at each time interval was reported to showcase how many participants remained eligible to experience the dropout event at each follow-up point.

Cox proportional hazards (PH) regression models were used to identify significant predictors of time to dropout. To maximize model parsimony and interpretability, potential redundancy and multicollinearity among candidate predictors were evaluated prior to regression modeling. Pairwise correlations among continuous variables were examined to identify highly overlapping measures. When multiple variables reflected the same underlying behavioral construct, a single representative measure was retained based on conceptual relevance and measurement consistency. Multicollinearity among retained predictors was further assessed using variance inflation factors (VIFs) computed from the predictor design matrix. VIF values for all retained predictors were found to be well below conventional thresholds, thus confirming the final model specification.

In all Cox models, time was parameterized as weeks since week 11. Models were estimated using (i) a counting-process (start–stop) formulation to allow for time-varying effects and (ii) the Efron method to handle tied event times. PH assumptions were evaluated using Schoenfeld residual diagnostics. Several engagement-related predictors demonstrated strong departures from proportional hazards, thus indicating time-dependent associations with dropout. Rather than excluding these predictors, we explicitly modeled non-proportionality where present. More precisely, workout frequency during the initial 28-day period (indexed by the number of active days) exhibited a clear time-varying association with adherence and was therefore modeled using an interaction with log(time), allowing its effect on dropout risk to attenuate over follow-up. Hazard ratios for workout frequency were subsequently evaluated at representative time points corresponding to early (week 11), mid (week 26), and later (week 40) follow-up, spanning the at-risk period while avoiding unstable tail estimates.

Age was included as a continuous covariate and modeled flexibly using restricted cubic splines (3 degrees of freedom) to accommodate non-linear associations with adherence. Considering that subscription uptake may be intertwined with early engagement and user motivation, and that both subscription status and sex exhibited strong departures from the PH assumption, these two variables were treated as stratification factors. Estimated associations should therefore be interpreted as conditional on subscription- or sex-related baseline hazard differences rather than as total causal effects.

### 2.7 Sensitivity analyses

A series of sensitivity analyses were conducted to assess the robustness of the primary findings to alternative operational definitions and measurement choices.

First, to evaluate whether results depended on the chosen adherence threshold, primary analyses were replicated using two stricter definitions of adherence in which either (i) no missed weeks were permitted during the observation period or (ii) users were required to complete at least two sessions per week over the course of twelve months following their initial workout. Results from this alternative allowance of intermittent lapses and required workout frequency are reported in the Supplementary Material (cf. Supplementary Figures S3–S6, and Supplementary Tables S4–S9).

Second, several engagement measures derived from the initial 28-day period—including workout duration and exercise-type composition—reflected moderately overlapping aspects of early engagement. To minimize instability in multivariable models, time-varying effects were modelled for one engagement measure at a time. Specifically, a series of Cox models was estimated in which each engagement variable was, in turn, included as the sole time-varying predictor in an otherwise identical model specification. Hazard ratios and 95% confidence intervals were extracted at representative timepoints, i.e., weeks 11, 26, and 40, to facilitate direct comparison across engagement operationalizations.

Third, to examine sensitivity to measurement errors arising from externally imported workouts—potentially noisier or systematically different—two complementary analyses were performed within the beginner cohort. In the first, primary models were re-estimated in a restricted sample of users who completed exclusively app-generated workouts during the initial 28-day period. In the second, models were re-estimated in the full beginner sample with additional adjustment for the proportion of workouts that were externally imported during the initial 28 days. These analyses were conducted to assess whether associations between early engagement behaviors and adherence were driven by differential logging practices rather than underlying exercise behavior. Results from both sensitivity analyses are reported in the Supplementary Material (cf. Supplementary Tables S10, S16).

### 2.8 Model interactions

To explore effect modifications by sex, age–dropout associations were examined within the Cox proportional hazards framework. Age was modeled flexibly using restricted cubic splines, thereby allowing the age effect to vary by sex. Model-based predicted hazard ratios for age were derived from the fitted models at a representative follow-up time (week 26), holding other covariates constant, and expressed relative to the cohort median age to facilitate interpretation and comparison across sexes. Formal evidence of effect modification was assessed using a joint likelihood ratio test comparing models with and without spline-based age × sex interaction terms.

To assess whether the association between early workout frequency (indexed by the number of active days during the initial 28-day period) and subsequent dropout risk varied as a function of workout duration, and whether this relationship changed over time, interaction effects between workout frequency and app-generated workout duration were also evaluated within Cox proportional hazards models. Both variables were treated as continuous predictors, and effect modification was assessed by incorporating reciprocal interaction terms, along with distinct interactions with log-transformed follow-up time. Formal evidence for effect modification and time-varying moderation was evaluated using likelihood ratio tests comparing nested models with and without these terms. To facilitate interpretation, model-based hazard ratios for workout frequency were subsequently derived at representative follow-up times (i.e., weeks 11, 26, and 40) and across representative levels of workout duration, holding other covariates constant.

### 2.9 Effect modification by experience level

To evaluate whether exercise experience level modified noteworthy associations between demographic, behavioral predictors, and long-term adherence, self-reported experience level was first incorporated into pooled Cox proportional hazards models including beginners, intermediate, and advanced users. To facilitate comparability of effect sizes across heterogeneous distributions and to improve numerical stability of time-varying interaction terms, continuous engagement variables were standardized (z-scored) prior to formal analysis. Model fits with and without experience level were compared using likelihood ratio tests to assess the overall contribution of experience to dropout risk. Proportional hazards assumptions were subsequently evaluated using Schoenfeld residual diagnostics. Given strong evidence that the effect of experience level varied over follow-up time, experience level was ultimately included as a stratification variable in pooled Cox models, allowing baseline dropout hazards to differ across experience groups while preserving estimation of other covariate effects.

To further characterize how experience level influenced adherence mechanisms, additional Cox regression analyses were conducted separately within each experience cohort. Specifically, demographic effect modification was examined using Cox models incorporating non-linear age effects and age × sex interactions, while behavioral effect modification was examined using models including interactions between early workout frequency and app-generated workout duration, as well as their time-varying associations with dropout risk. Results from these cohort-specific models were subsequently compared to identify similarities and differences in adherence patterns across experience levels.

## 3 Results

### 3.1 User sample characteristics

The final sample retained for the present study included 389,481 users. Table 1 provides a comprehensive summary of users' basic demographic characteristics and corresponding descriptive statistics (means and standard deviations or counts and percentages, as appropriate), stratified by experience level. The proportion of male users was found to steadily increase with self-reported experience level, while age distributions were broadly comparable across beginner, intermediate, and advanced users. More specifically, for the beginner cohort, 100,709 users were retained in the final dataset. Of these, 62.2% were males and 37.8% were females. Approximately three quarters of the sample (75.8%) were between 18 and 40 years with a mean age of 34 years. For the intermediate cohort, 193,621 users were retained in the final dataset. Of these, 69.8% were males and 30.2% were females. Approximately three quarters of the sample (74.4%) was between 18 and 40 years with a mean age of 34.5 years. For the advanced cohort, 95,151 users were retained in the final dataset. Of these, 78.7% were males and 21.3% were females. Approximately two thirds of the sample (70.2%) was between 18 and 40 years with a mean age of 35.9 years.

**Table 1:** Users’ demographic characteristics and corresponding descriptive statistics, stratified by self-reported experience level.

Characteristic	Beginner	Intermediate	Advanced
Sex			
Female	38,027 (37.8%)	58,388 (30.2%)	20,263 (21.3%)
Male	62,682 (62.2%)	135,233 (69.8%)	74,888 (78.7%)
Age			
Mean ± SD	34.07 ± 10.07	34.52 ± 9.84	35.87 ± 9.60
Age groups			
18–30	42,314 (42.0%)	75,643 (39.1%)	30,483 (32.0%)
31–40	34,043 (33.8%)	68,403 (35.3%)	36,361 (38.2%)
41–50	17,168 (17.0%)	36,033 (18.6%)	20,907 (22.0%)
51–60	5,708 (5.7%)	11,188 (5.8%)	6,269 (6.6%)
61–70	1,291 (1.3%)	2,120 (1.1%)	1,039 (1.1%)
71–80	175 (0.2%)	226 (0.1%)	88 (0.1%)
81+	10 (0.01%)	8 (0.003%)	4 (0.004%)

Table 2 presents descriptive statistics for early exercise behaviors in the beginner cohort, reported as means and standard deviations for continuous variables, and counts and percentages for categorical variables. These data characterize users' training frequency, workout composition, equipment use, and subscription patterns during the initial 28 days of app engagement. The mean and median total workout times during the first 28 days were found to be approximately 1,017 and 812 min (IQR: 565–1,191), respectively, the latter corresponding to roughly 13.5 h of exercise per month, or about 3.5 h per week. Of this, approximately 719 (mean) and 568 (median) minutes (IQR: 328–885) were completed via app-generated workouts, the latter being equivalent to about 9.5 h per month. These values indicate that beginners typically relied on the app as their main training modality (≈71% of recorded workout time), while engaging in an additional 4–5 h of externally sourced activities on average. Of these externally imported sessions, which were typically shorter and represent approximately 40% of the total number of workout sessions completed, the large majority originated from Apple Health (33%), with smaller contributions from FitBit (4%) and Strava (2%).

**Table 2:** Descriptive statistics for early exercise behaviors in the beginner cohort.

Early exercise behaviors in beginner users	*N* = 100,709^a^
Total workouts completed in the first 28 days	21 ± 13
App workouts completed in the first 28 days	12 ± 6
Total workout time (min) in the first 28 days	1,017 ± 749^b^
App workout time (min) in the first 28 days	719 ± 666^c^
% of workouts including mobility	13 ± 23
% of workouts including cardio	47 ± 38
% of strength-based exercises	77 ± 24
Days with at least one workout (first 28 days)	15 ± 4
Days with app strength work	11 ± 5
Days with mobility work	2 ± 4
Days with cardio work	8 ± 7
Distinct equipment types used	14 ± 10
Paid app subscription	77,488 (77%)

a Data presented as Mean ± SD or *N* (%).

b Total workout duration (winsorized): Median: 812 min; IQR: 565–1,191 min.

c App workout duration (winsorized): Median: 568 min; IQR: 328–885 min.

### 3.2 Survival analysis

**Figure 1:**
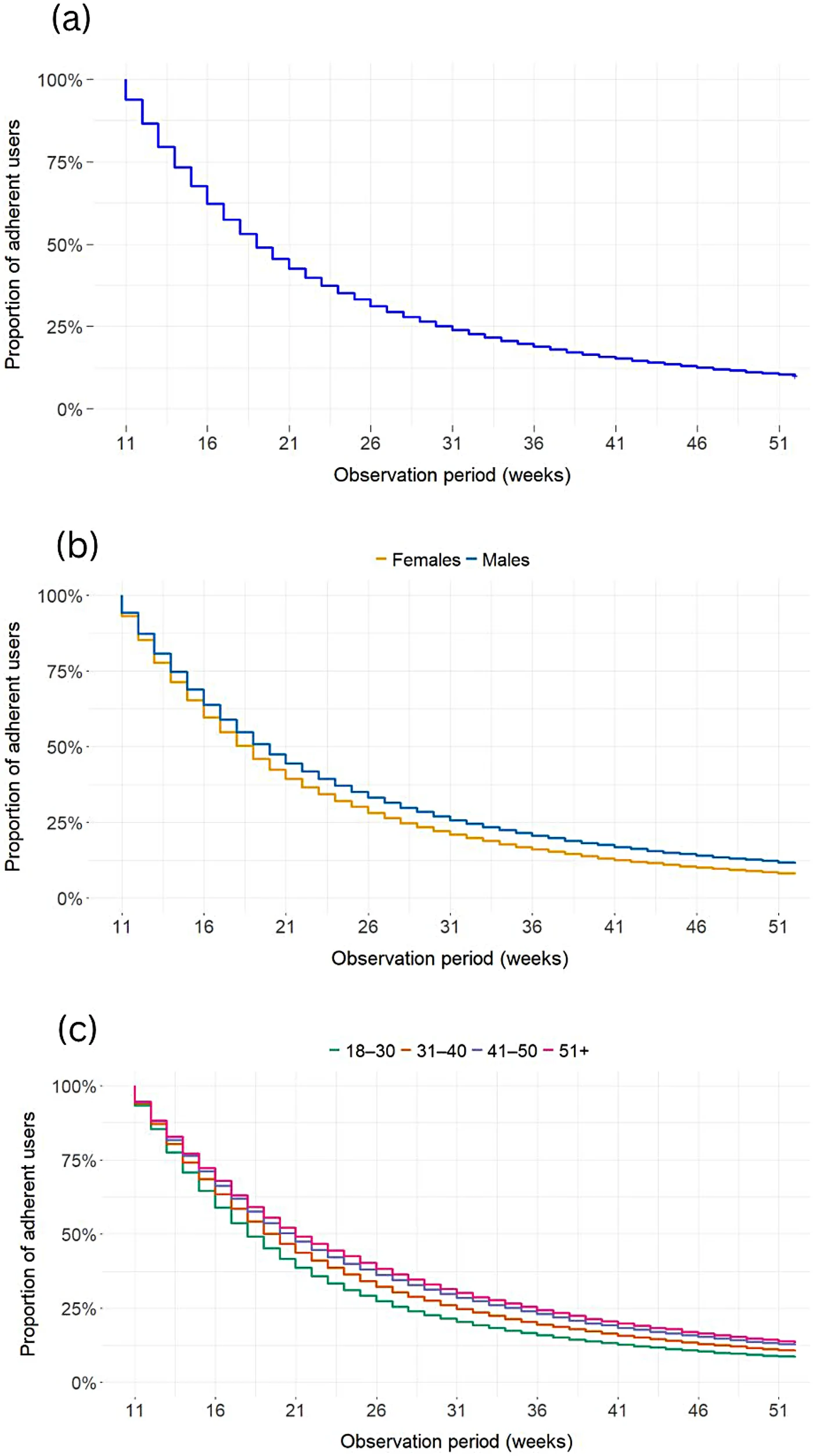
Kaplan–meier survival curves for the beginner cohort. Estimated proportion of users from the entire beginner cohort who remained adherent during the observation period **(a)** and stratified by sex **(b)** and age group **(c)** The Kaplan–Meier curves are displayed from week 11 onward, reflecting the earliest time point at which users could meet the study definition of non-adherence, given the requirement of at least one workout per week during the initial 28-day onboarding period and allowance for up to six weeks with no active days. Adherence declined steadily over time, with a median time to dropout of 19 weeks and an estimated 10.1% of users remaining adherent at week 52. Male beginners and users aged 51 and older showed a modestly higher probability of adherence throughout the observation period.

Figure 1a presents the Kaplan–Meier survival curves describing adherence over the observation period in the beginner cohort. Adherence declined steadily across time, with the steepest decrease occurring between weeks 10 and 15. The median time to dropout was 19 weeks, indicating that half of all beginners ceased meeting adherence criteria by this point. After approximately 26 weeks, the curve began to gradually flatten, suggesting the emergence of a smaller subset of users who maintained consistent training behavior throughout the full follow-up period. By week 52, the estimated probability of remaining adherent was 10.12%, corresponding to approximately 10,192 beginner users who met the study long-term adherence definition.

Figure 1b presents separate Kaplan–Meier survival curves for males and females within the beginner cohort. While adherence declined across both groups, males consistently exhibited a higher probability of remaining adherent throughout follow-up (cf. Supplementary Table S1). A log-rank test confirmed that the survival distributions differed significantly between sexes (*χ*^2^ = 463, *p* < 0.001). By week 52, the estimated adherence probability was 7.95% for females and 11.43% for males.

Kaplan–Meier curves stratified by age revealed marked differences in adherence trajectories across the observation period (cf. Figure 1c). Younger adults (18–30 years) showed the steepest decline, whereas adherence progressively improved with increasing age (cf. Supplementary Table S1). A log-rank test confirmed significant differences in survival distributions across age groups (*χ*^2^ = 755, *p* < 0.001). At week 52, estimated adherence probabilities were 8.37% for users aged 18–30 years, 10.45% for those aged 31–40, 12.44% for those aged 41–50, and 13.31% for users aged 51 years and older.

A comprehensive summary of the proportion of beginner users at risk of dropout over the course of follow-up, stratified by sex and age can be found in Supplementary Table S1.

### 3.3 Cox proportional hazards model

Predictor redundancy and collinearity were evaluated via inspection of both correlation coefficients and variance influence factors (cf. Supplementary Figure S2 and Supplementary Table S2). Associations between retained early exercise behaviors and subsequent loss of adherence were examined using separate Cox proportional hazards models with time-varying effects.

In the primary model, workout frequency during the initial 28-day period was strongly associated with subsequent loss of adherence (likelihood ratio *χ*^2^ = 3,050.35, *df* = 1, *p* < 0.001). This association varied significantly over time [interaction with log(time): *χ*^2^ = 295.44, *df* = 1, *p* < 0.001], indicating attenuation of the protective effect as follow-up progressed. Specifically, each standard deviation increase in the number of active days was associated with a 27% lower hazard of dropout at week 11 (HR = 0.73, 95% CI: 0.72, 0.74; cf. Supplementary Table S3). This association weakened but remained substantial at week 26 (HR = 0.87, 95% CI: 0.86, 0.88) and week 40 (HR = 0.91, 95% CI: 0.90, 0.92).

Age was significantly associated with adherence in a non-linear fashion (*χ*^2^ = 649.46, *df* = 3, *p* < 0.001) and was modelled using restricted cubic splines. Sex and subscription status were included as stratification factors.

These findings were robust to both alternative adherence definitions and restriction of the analysis to app-generated workouts only, with nearly identical effect sizes and temporal attenuation patterns observed (cf. Supplementary Figures S3–S6, and Supplementary Tables S4–S9).

### 3.4 Sensitivity analyses

Sensitivity analyses were used to examine moderately overlapping operationalizations of early engagement by modelling each measure as the sole time-varying predictor in otherwise identical Cox models. Across all measures considered, higher levels of early engagement (i.e., greater percentage of strength-based workouts, longer workout durations, and greater equipment diversity) were consistently associated with lower dropout risk early in follow-up, with effect sizes attenuating over time (cf. Supplementary Table S11).

**Figure 2:**
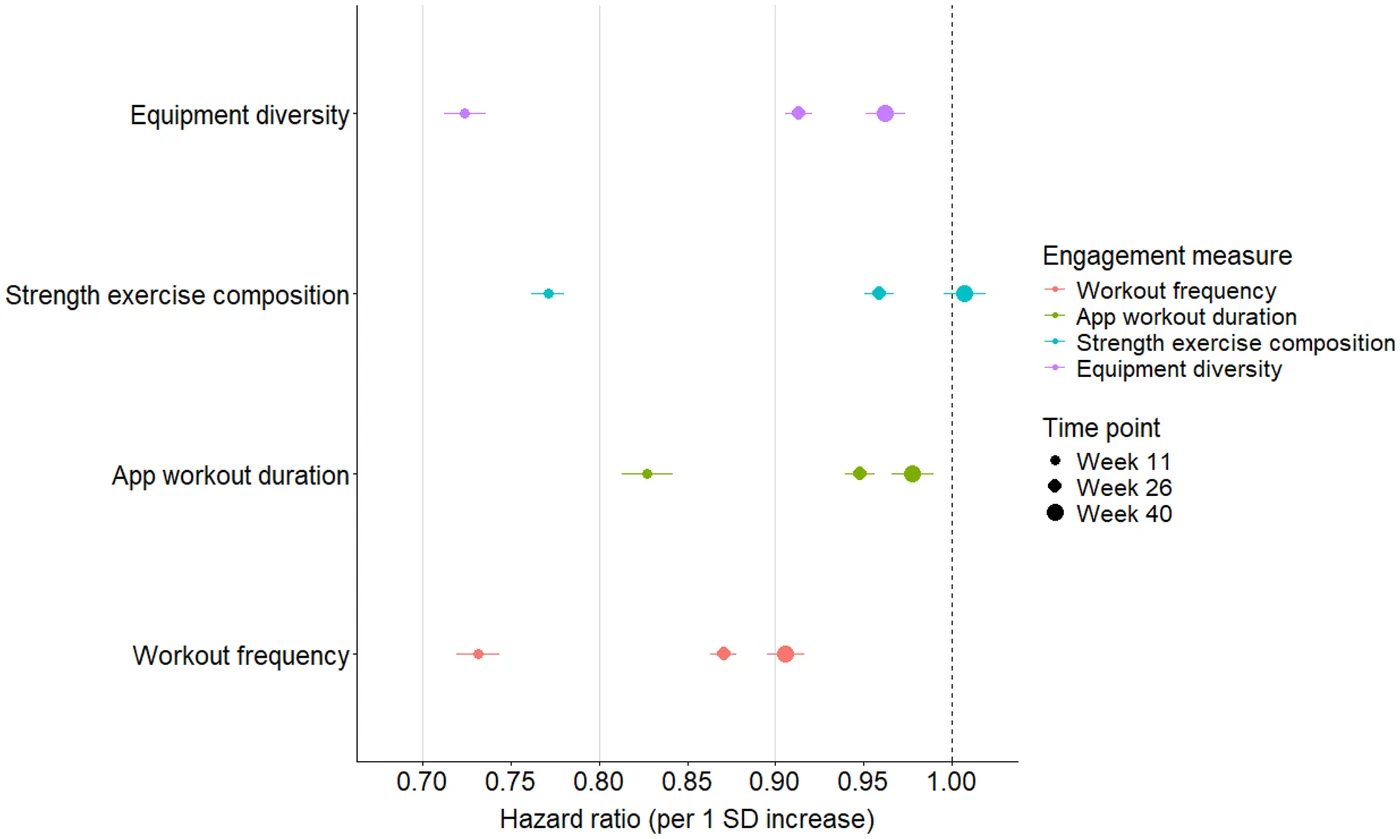
Time-dependent hazard ratios for early engagement behaviors. Points indicate hazard ratios (per one standard deviation increase) evaluated at weeks 11, 26, and 40; bars indicate 95% confidence intervals. Dotted lines connect estimates for the same engagement measure across time to illustrate attenuation of detected associations over time. Age was modeled with restricted cubic splines (3 degrees of freedom). All models were stratified by sex and subscription status.

For example, greater app-generated workout duration during the initial 28-day period was associated with a 17% lower dropout hazard at week 11 (HR = 0.83, 95% CI: 0.81, 0.84), attenuating to HR = 0.95 (95% CI: 0.94, 0.96) at week 26 and HR = 0.98 (95% CI: 0.97, 0.99) by week 40. A similar pattern was observed for both equipment diversity and the proportion of strength-based exercises whereby protective associations became progressively weaker throughout follow-up (cf. Figure 2).

### 3.5 Interaction effects

To explore potential effect modifications contingent on demographic characteristics, we first evaluated whether key associations, we assessed whether the relationship between age and dropout risk varied by sex.

**Figure 3:**
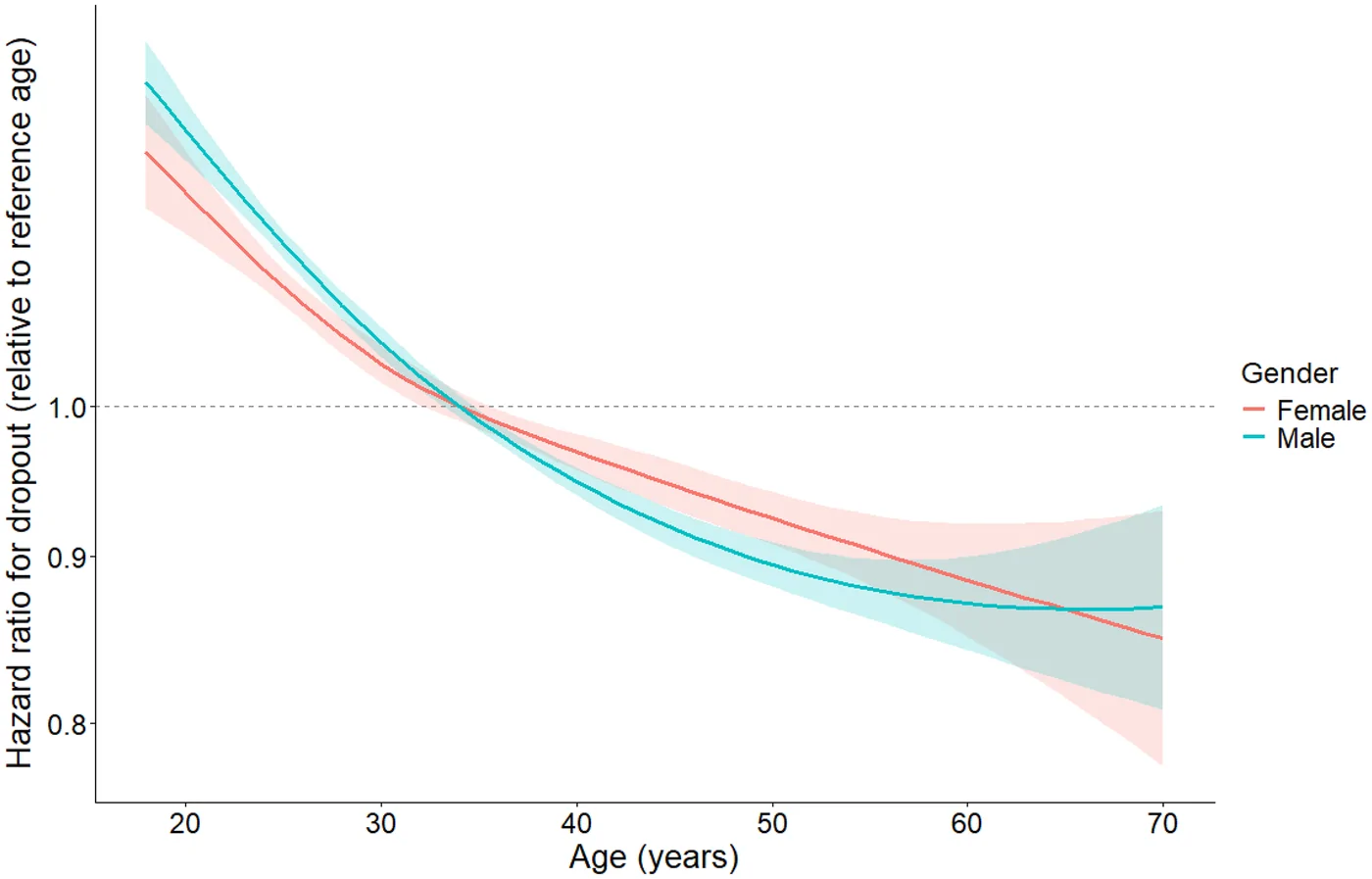
Age–dropout associations by sex. Predicted hazard ratios for dropout as a function of age, shown separately for female and male users, derived from sex-specific Cox proportional hazards models. Hazard ratios are expressed relative to the cohort median age and are evaluated at a representative follow-up time (week 26). Models were adjusted for early workout frequency with a time-varying effect and stratified by subscription status. Shaded bands represent 95% confidence intervals.

In Cox proportional hazards models with age modeled flexibly using restricted cubic splines, a joint likelihood ratio test comparing models with and without spline-based age × sex interaction terms provided evidence of effect modification by sex [*χ*^2^(3) = 13.32, *p* = 0.004]. To illustrate this effect, Figure 3 displays model-based predicted hazard ratios for age, evaluated at a representative follow-up time (week 26) and expressed relative to the cohort median age (33 years), separately for female and male users. Consistent with these curves, age-specific hazard ratios derived from the fitted model indicated that, relative to age 33, dropout risk at age 25 was higher for both females (HR = 1.08, 95% CI: 1.06–1.10) and males (HR = 1.11, 95% CI: 1.10–1.12), whereas dropout risk at ages 40 (females: HR = 0.96, 95% CI: 0.95–0.98; males: HR = 0.94, 95% CI: 0.93–0.95) and 55 was lower (females: HR = 0.90, 95% CI: 0.87–0.93; males: HR = 0.87, 95% CI: 0.85–0.89). Younger users exhibited higher dropout risk relative to the median age regardless of sex, with risk declining across early and middle adulthood. However, among women this decline was approximately monotonic, whereas among men the association reached a minimum in late middle age (66.4 years, HR = 0.86) and increased modestly at older ages. Despite these differences in curvature, the overall magnitude of age-related effects was similar across sexes, with substantial overlap between the predicted curves.

Next, we examined whether workout frequency and app-generated workout duration exhibited a significant interactive association with dropout risk. A likelihood ratio test comparing models with and without the workout frequency × duration interaction indicated strong evidence of effect modification [*χ*^2^(1) = 84.67, *p* < 0.001], with further model comparisons supporting time-varying moderation of this interaction over follow-up [*χ*^2^(1) = 33.01, *p* < 0.001]. In the fully adjusted Cox model, workout frequency showed a strong protective association early in follow-up (i.e., week 11; *β* = −0.32, HR = 0.73, *p* < 0.001, cf. Supplementary Table S12), an effect found to attenuate over time [workout frequency × log(time): *β* = 0.07, HR = 1.07, *p* < 0.001]. Importantly, the protective effect of workout frequency was stronger among users completing longer app-generated workouts, as reflected by a significant frequency × duration interaction (*β* = −0.08, HR = 0.93, *p* < 0.001). This duration-based moderation was also found to diminish over time [workout frequency × duration × log(time): *β* = 0.02, HR = 1.02, *p* < 0.001]. Consistent with these effects, time-specific hazard ratios derived from the fitted model indicated that a one–standard deviation increase in workout frequency was associated with a 33% reduction in dropout risk at week 11 among users completing longer workouts, compared with a 21% reduction among users completing shorter workouts, with differences progressively reducing over time (Table 3).

**Table 3:** Time-specific hazard ratios for a 1-SD increase in workout frequency across levels of app-generated workout duration.

Duration	HR (Week 11)	HR (Week 26)	HR (Week 40)
Short (−1 SD)	0.79 [0.77–0.80]	0.89 [0.88–0.91]	0.92 [0.90–0.94]
Average	0.73 [0.72–0.74]	0.88 [0.87–0.89]	0.92 [0.91–0.93]
Long (+1 SD)	0.67 [0.66–0.69]	0.86 [0.85–0.87]	0.91 [0.90–0.93]

Hazard ratios (HRs) and 95% confidence intervals quantify the association of a one–standard deviation increase in workout frequency with dropout risk, conditional on app-generated workout duration. Estimates are derived from the fitted time-varying Cox proportional hazards model and evaluated at weeks 11, 26, and 40 of follow-up. All models adjust for age (modeled using restricted cubic splines), sex, and are stratified by subscription status.

### 3.6 Sensitivity analysis of adherence definition

To assess the robustness of our main findings to the operational definition of long-term adherence, we conducted two sensitivity analysis using stricter criteria in which either no missed weeks were permitted during the observation period, or users were required to complete at least two workouts per week. These analyses were performed within the beginner cohort and replicated the primary Cox modeling framework. Specifically, we re-estimated models examining (i) the age × sex interaction using spline-based age terms and (ii) the interaction between workout frequency and app-generated workout duration, including their time-varying effects. Overall, the direction, relative magnitude, and temporal pattern of associations observed under the original adherence definition were preserved under both stricter criteria. In particular, age continued to exhibit a non-linear association with dropout risk that differed modestly by sex, and higher early workout frequency remained most strongly protective when paired with longer app-generated workouts, with effects attenuating over follow-up. Detailed results from these analyses are presented in the Supplementary Material (cf. Supplementary Figures S3–S6, and Supplementary Tables S4–S9).

### 3.7 Effect of experience level on long-term adherence

Kaplan–Meier curves on the pooled sample stratified by experience level revealed marked differences in adherence trajectories across the observation period (cf. Supplementary Figure S7). Greater training experience appeared to be protective against dropout risk, and increasingly so. At week 52, estimated adherence probabilities were 10.1% for beginners, 18.3% for intermediate users, and 25.8% for advanced users (cf. Supplementary Table S13). The log-rank test confirmed significant differences in survival distributions across these groups (*χ*^2^ = 14,282, *p* < 0.001).

In pooled Cox proportional hazards analyses including beginners, intermediate, and advanced users, experience level was strongly associated with dropout risk. A likelihood ratio test comparing models with and without experience level indicated a substantial improvement in model fit when experience was included [*χ*^2^(2) = 10,588 *p* < 0.001]. However, proportional hazards diagnostics revealed that the effect of self-reported experience varied over follow-up time. Accordingly, this variable was stratified in the final pooled Cox model (cf. Supplementary Table S14), allowing baseline dropout hazards to differ across experience groups while preserving estimation of demographic and behavioral covariate effects. Consistent with results observed in the beginner cohort, higher early workout frequency was strongly associated with lower dropout risk, with a one–standard deviation increase corresponding to a 26% reduction in hazard at the beginning of follow-up (HR = 0.74, 95% CI: 0.74–0.75). This protective association attenuated over time, as reflected by a positive interaction with log-transformed follow-up time [HR = 1.05 per unit increase in log(time), 95% CI: 1.05–1.06]. Once again, age exhibited a pronounced non-linear association with dropout risk, and male users had a lower hazard of dropout compared with female users (HR = 0.86, 95% CI: 0.86–0.87).

To further characterize whether demographic determinants of adherence differed by exercise experience, separate Cox proportional hazards models were estimated within beginner, intermediate, and advanced user cohorts. Age was modeled flexibly using restricted cubic splines and its interaction with sex was formally tested to assess sex-specific differences in the age–dropout association.

As previously discussed, the joint likelihood ratio test comparing models with and without spline-based age × sex interaction terms provided evidence of effect modification by sex in the beginner cohort [*χ*^2^(3) = 13.53, *p* = 0.004]. Similar evidence of sex-dependent age effects was observed among intermediate users [*χ*^2^(3) = 52.09, *p* < 0.001] and advanced users [*χ*^2^(3) = 53.74, *p* < 0.001]. Across all experience levels, these results indicate that the association between age and dropout risk differs systematically between females and males, although the magnitude of this interaction varies by cohort.

**Figure 4:**
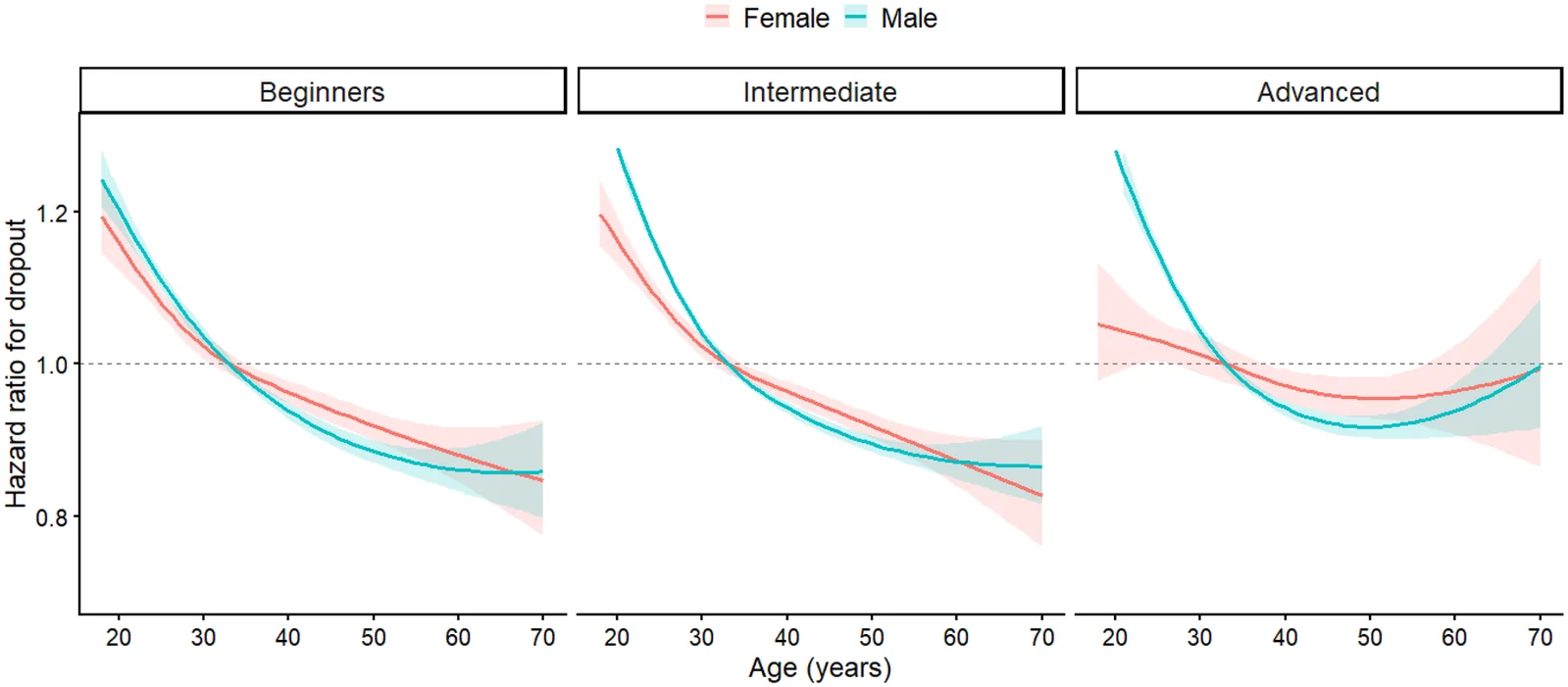
Sex-specific age–dropout associations by experience level. Model-based predicted hazard ratios for dropout as a function of age, shown separately for females and males within beginner, intermediate, and advanced user cohorts. Estimates were derived from Cox proportional hazards models in which age was modeled using restricted cubic splines with an age × sex interaction, evaluated at week 26 of follow-up. Hazard ratios are expressed relative to the cohort median age (33 years). Models adjust for workout frequency and are stratified by subscription status. Shaded bands represent 95% confidence intervals.

Model-based predicted hazard ratios illustrating sex-specific age patterns within each experience group are presented in Figure 4, with corresponding age-specific estimates reported in Supplementary Table S15. Relative to the cohort median age (33 years), younger users exhibited higher dropout risk across all experience levels, and this effect was consistently more pronounced among males than females. Notably, among advanced users, the hazard of dropout at age 25 was 4% higher for females (HR = 1.04, 95% CI: 1.01–1.07) and 17% higher for men (HR = 1.17, 95% CI: 1.16–1.18), whereas older ages were associated with progressively lower dropout risk (HR = 0.97 and HR = 0.94, respectively). These estimates quantify the non-linear and sex-dependent association between age and adherence and demonstrate that such patterns persist across levels of exercise experience.

Lastly, we estimated cohort-specific Cox proportional hazards models testing interactions between workout frequency and app-generated workout duration, as well as their time-varying associations with dropout risk. Replicating the pattern of results observed among beginner users in the intermediate cohort, models including the frequency × duration interaction demonstrated improved fit [*χ*^2^(1) = 103.92, *p* < 0.001], while also providing strong evidence of time-varying moderation [*χ*^2^(1) = 37.45, *p* < 0.001, cf. Table 4]. Higher workout frequency conferred greater early protection against dropout among users completing longer workouts, with effect sizes converging across duration levels later in follow-up.

**Table 4:** Time-specific hazard ratios for workout frequency by workout duration and experience level.

Experience level	Duration	HR (Week 11)	HR (Week 26)	HR (Week 40)
Beginner	Short (−1 SD)	0.82 [0.80–0.83]	0.90 [0.88–0.91]	0.92 [0.90–0.93]
	Average	0.75 [0.74–0.77]	0.89 [0.87–0.90]	0.92 [0.90–0.93]
	Long (+1 SD)	0.69 [0.68–0.71]	0.87 [0.86–0.89]	0.92 [0.90–0.94]
Intermediate	Short (−1 SD)	0.79 [0.77–0.80]	0.88 [0.86–0.89]	0.90 [0.88–0.91]
	Average	0.74 [0.73–0.75]	0.86 [0.85–0.87]	0.89 [0.88–0.90]
	Long (+1 SD)	0.69 [0.68–0.71]	0.84 [0.83–0.86]	0.88 [0.87–0.90]
Advanced	Short (−1 SD)	0.77 [0.74–0.79]	0.86 [0.84–0.88]	0.88 [0.86–0.90]
	Average	0.73 [0.71–0.75]	0.85 [0.83–0.86]	0.88 [0.86–0.89]
	Long (+1 SD)	0.69 [0.67–0.72]	0.84 [0.82–0.85]	0.87 [0.85–0.89]

Hazard ratios (HRs) represent the association between a one–standard deviation increase in workout frequency and dropout risk, evaluated at weeks 11, 26, and 40 of follow-up, at short (−1 SD), average (0 SD), and long (+1 SD) app-generated workout durations. Estimates are derived from cohort-specific Cox models including frequency × duration and frequency × duration × log(time) interaction terms and are adjusted for age and sex, with stratification by subscription status.

By contrast, among advanced users, evidence for moderation by workout duration was present but less pronounced. The inclusion of the frequency × duration interaction improved model fit [*χ*^2^(1) = 31.47, *p* < 0.001], and the magnitude of the corresponding time-varying interaction was comparatively smaller [*χ*^2^(1) = 12.3, *p* < 0.001]. Taken together, these findings indicate that the sustainability benefits of pairing frequent training with longer app-generated workouts are strongest among less experienced users and are most pronounced early in the adherence trajectory.

## 4 Discussion

The present study leveraged large-scale, objectively recorded exercise data to examine predictors of long-term exercise adherence across the adult lifespan, in both sexes, and at varying levels of training experience, thereby addressing important gaps within the relevant scientific literature. Prior research on fitness interventions using smartphone apps has consistently highlighted the challenges of long-term engagement, with high attrition rates and limited sustained use observed in both predictive and empirical studies of mobile fitness platforms (21). Systematic reviews further underscore the lack of consistent adherence measurement and the need for objective, longitudinal data to better identify reliable predictors of sustained engagement (22).

Building on the existing evidence-based knowledge, and by combining time-to-event survival analyses with detailed characterization of early training behaviors, we provide evidence that early consistency, structured engagement, and training context play a central role in sustaining exercise participation over time in ecologically valid settings. Importantly, these associations proved robust across demographic strata while varying systematically with users' self-reported experience level and over the course of follow-up.

Among beginners, adherence declined rapidly following onboarding, with a median time to dropout occurring at 19 weeks and approximately 10% of users remaining adherent at twelve months. Such high attrition rate is consistent with prior research, where patterns of early drop off, particularly among digital app users, are well documented (23, 24). Perhaps not surprisingly, adherence improved monotonically as self-reported experience increased, with intermediate and advanced users exhibiting progressively higher retention throughout the observation period and at six months (18.3% and 25.8%, respectively). Within the beginner cohort, adherence trajectories further differed systematically by demographic factors in that older users and male users showed slower declines in exercise participation. These patterns highlight that early disengagement is common, particularly among novice users, but that both prior experience and contextual support meaningfully shape the likelihood of sustained participation over time.

Across all analyses performed, early training consistency (i.e., during the first 28 days) emerged as the strongest predictor of sustained app-recorded exercise adherence. A greater number of active training days during onboarding was consistently associated with a substantially lower risk of dropout, even after accounting for non-proportional hazards through explicit modeling of time-varying effects. This protective association was evident early in follow-up and attenuated gradually over time, suggesting that early consistency may play a particularly critical role in establishing sustainable exercise habits, possibly by fostering greater tangible progress, which might in turn increase self-confidence and enhance motivation. These findings directly corroborate theoretical models of habit formation, which emphasize behavioral regularity rather than sheer training volume as a primary driver of successful long-term behavior maintenance (25). From a practical standpoint, these temporal patterns suggest that the greatest opportunity to improve long-term engagement lies during the earliest stages of app use. For fitness professionals and coaches, encouraging clients to establish a sustainable weekly routine may be more beneficial than prescribing highly ambitious training schedules that prove difficult to maintain. Likewise, digital fitness platforms may benefit from designing onboarding experiences that reinforce early consistency through progressive goal setting, reminders, adaptive programming, and feedback mechanisms that reward regular participation rather than exercise volume alone.

In parallel, recent tablet-supported home exercise interventions, such as the VITAMIN study, suggest that early app usage frequency predicts subsequent exercise adherence (26). By leveraging a substantially larger, non-clinical sample and objective workout logs collected over several months, the present study extends these insights into real-world commercial fitness app settings and complements smaller, controlled or laboratory-based studies [e.g., GPTCoach, Jörke, Sapkota (27); Tele-PhyT, Conroy, Brunetti (28)], which have primarily focused on intervention design and short-term behavioral outcomes rather than longitudinal adherence trajectories.

The observed frequency effect attenuation over follow-up is also consistent with prior research on digital engagement trajectories, which reveals that app use often unfolds through cycles of disengagement and re-engagement rather than being captured as a single behavioral episode followed by irreversible dropout (29).

The interaction between early training consistency and workout duration clarified that longer sessions were not inherently protective. Instead, longer app-generated workouts were associated with improved adherence primarily among users who trained more frequently, particularly during the early stages of follow-up, suggesting that session duration may serve as a marker of sustained engagement or exercise commitment rather than an independent driver of adherence. In other words, users who successfully established a consistent exercise routine may have been more willing or able to complete longer sessions as their habits became established, whereas longer workouts alone did not appear sufficient to offset the negative consequences of inconsistent participation. These findings therefore suggest that training frequency and workout duration should be viewed as complementary rather than competing behavioral characteristics during the early stages of exercise adoption. This pattern further indicates that extended training sessions may enhance adherence when embedded within a regular routine but may be less beneficial, or even counterproductive, in the absence of consistent engagement. Together, these findings reinforce the notion that habit formation and routine integration—rather than maximal effort per session—most likely underpin long-term exercise adherence and support onboarding strategies that first encourage regular participation and subsequently progress session duration as exercise habits become established.

As previously mentioned, greater diversity in early exercise exposure was also associated with lower dropout risk. Users who engaged with a broader range of equipment types and exhibited stronger emphasis on strength-based exercise during the onboarding period were more likely to sustain app-recorded exercise participation over time. Although these measures were partially correlated with overall engagement, sensitivity analyses indicated that their associations were directionally consistent across model specifications. The magnitude of this protective effect, however, was found to progressively decrease throughout the observation period. It is plausible that early variation in training stimuli enhances engagement by promoting skill acquisition, encouraging progressive overload, and improving perceived competence, thereby reducing monotony and the likelihood of disengagement (30), particularly in the early stages of exercise participation.

With respect to demographic determinants, both age and sex were independently associated with dropout risk, with older users and male users exhibiting lower hazards of disengagement overall. Importantly, however, while these associations were highly statistically significant, their practical magnitude was comparatively modest relative to the behavioral predictors identified in this study. Flexible spline-based modeling pointed to non-linear associations between age and adherence that differed modestly by sex. Specifically, the protective association of increasing age was more pronounced among men, especially during mid-to-late adulthood. Although the mechanisms underlying this pattern are not fully elucidated, differences in training history, motivational structure, and perceived barriers to exercise across sexes and age groups may be involved (31–33). For example, observational studies using population data consistently reveal that men have greater participation in resistance training compared to women (34). Considering that fitness apps like the one employed in this study are mostly used to generate and access resistance training programs, and that app engagement was considered as a proxy for long-term adherence, such disparity between sexes in physical activity patterns may have contributed to the higher likelihood of sustained exercise participation observed in males compared to females. Besides, field deployments of digital exercise interventions targeting older adults have reported heterogeneous engagement patterns and usability challenges [e.g., Senior Fit, Lee, Hyun (35)]. Within this context, the present finding of lower dropout risk among older users may appear counterintuitive. However, it may simply reflect differences in sampling frames: whereas prior studies often include clinically referred or technology-naïve populations, the present cohort comprises self-selected app users who already possess baseline digital knowledge. Within such a motivated population, older adults may be expected to exhibit greater consistency, potentially due to more stable routines, health-oriented goals, or fewer competing time demands. Our results therefore nuance—rather than contradict—existing evidence on age-related engagement in digitally-driven exercise practices.

Extending beyond the beginner cohort, pooled analyses incorporating experience level as an additional predictor demonstrated significant differences in dropout risk, with intermediate and advanced users exhibiting lower hazards of disengagement throughout the observation period. Follow-up cohort-specific analyses further revealed that the core associations observed among beginners—including the time-varying protective effect of early training consistency and its interaction with workout duration—were largely preserved across experience levels, albeit with some differences in magnitude. The protective effect of early consistency strengthened with increasing experience, suggesting that more experienced exercisers may be better equipped to translate early behavioral regularity into long-term habits through greater self-efficacy, goal clarity, and tolerance for training demands.

From a methodological perspective, this study has several notable strengths. These include a very large sample size, the use of objectively recorded behavioral data, the longitudinal follow-up, and the application of survival modelling to characterize adherence dynamics over time. Unlike much of the prior literature, which has relied primarily on self-reported measures, short-term interventions, and narrowly defined or clinical populations (38–40), the present analyses capture real-world exercise behaviors across a heterogeneous user base and a broad range of training contexts. Importantly, the primary findings were robust across multiple sensitivity analyses, including including substantially stricter operational definitions of adherence (no missed weeks permitted or a minimum of two workouts per week), restrictions to app-generated workouts only, and model adjustments for the proportion of externally imported workouts, indicating that the observed associations were not driven by specific analytic thresholds or measurement choices. Because of the very large sample size, statistical tests were highly powered to detect even small differences. Accordingly, we interpreted findings primarily according to their effect sizes, temporal patterns, and practical relevance rather than *p*-values or likelihood-ratio statistics alone. Together, these results provide ecologically valid insights into early behavioral factors associated with sustained exercise engagement outside controlled research settings.

Several limitations should also be acknowledged. First, although detailed information on training frequency, duration, and exercise composition was captured via the app interface, users may have engaged in additional workout sessions that were not subsequently documented within the app, potentially leading to an underestimation of overall exercise exposure. Second, and following from the previous point, app disengagement may not truly reflect failure in exercise adherence. Users who dropped out based on the study predefined criteria may have continued engaging in other forms of physical activity external to the app environment or simply ceased to log their workouts. In such cases, the primary outcome measure may have misclassified true exercise behavior. This source of misclassification may be particularly relevant among more experienced users, who may gradually become less reliant on structured app guidance as exercise routines become established or transition to alternative tracking platforms or self-directed training.

Related considerations concern the restriction of all analyses to users who demonstrated minimal engagement during the onboarding period (i.e., completed at least one workout per week during the first 28 days of app use). However, users who disengaged immediately after initial registration provided insufficient exercise exposure to meaningfully characterize the effect of training frequency, duration, or composition on long-term adherence, rendering exercise-related predictors largely uninformative. In this context, early disengagement (i.e., <4-weeks) likely reflects heterogeneous factors unrelated to training behavior *per se* (e.g., usability issues, unmet expectations, or contextual barriers) rather than responses to specific exercise patterns. Accordingly, the analytic focus on users who engaged during the first 28 days was necessary to isolate variation in early training behaviors and examine how these behaviors relate to subsequent adherence trajectories. The findings should therefore be interpreted as characterizing determinants of sustained engagement among initially active users, rather than predictors of initial app adoption or immediate disengagement in the broader population of new app users.

Third, estimated workout durations did not take into account rest periods between sets, which may vary to a considerable extent based on numerous factors, such as user preferences, restricted (or extended) time availability to complete the exercise session, and exercise intensity, yet another variable that was not included in the current dataset. Further enquiries controlling for these interrelated components or looking at finer-grained metrics like training load (i.e., number of sets and reps, and relative weight used) will be necessary to complement and corroborate our findings while keeping in mind that behaviors observed in any cohort of digital app users may not entirely reflect those of the general population at large.

Fourth, the present analyses did not explicitly adjust for calendar-time effects such as seasonality, secular trends in app use, or disruptions related to the COVID-19 pandemic, which may have induced significant changes to workout engagement patterns. We elected not to condition our user selection on calendar time to maximize sample size and statistical power and to preserve comparability across cohorts recruited over a long observation window spanning several years.

More broadly, several potentially relevant psychosocial factors—such as motivation, social support, training goals, or injury history—were not available and therefore could not be accounted for in our statistical plan, yet evidence suggests they may facilitate positive attitude changes in relation to exercise (41). For example, recent work suggests that app features supporting accountability—such as peer comparison, shared goals, or group-based challenges—personalization, and gamification may influence ongoing app use and reinforce regular training habits (42), though long-term adherence remains largely under-studied in real-world settings (43). Related to this point, an additional factor that may influence long-term exercise adherence is the training environment. Home- and gym-based exercise differ with respect to accessibility, equipment availability, social context, and logistical barriers, all of which may influence long-term engagement (36). Although the present analyses incorporated measures of available equipment and exercise diversity, the dataset did not contain a direct indicator of whether users primarily trained at home or in a gym. Consequently, the independent contribution of training environment could not be evaluated and represents an important direction for future research.

Importantly, all analyses performed were strictly observational in nature, and the reported associations should therefore not be interpreted as causal. Early training behaviors were operationalized using data from users' first 28 days of app use and treated as baseline predictors; although their effects were allowed to vary over follow-up time, changes in training patterns beyond this initial window were not explicitly modeled and may have influenced subsequent adherence. Finally, experience level was self-reported; although it is reasonable to assume that users identifying as beginners (<1 year of experience) genuinely belonged to this category, the criteria used by individuals to self-classify as intermediate or advanced may have varied, potentially introducing heterogeneity within these groups.

These considerations aside, the present findings offer valuable insights for the design of exercise programs, digital fitness platforms, and public health interventions, while also highlighting important avenues for future research incorporating richer contextual and psychosocial information. Frequent training sessions during the onboarding phase were consistently associated with improved long-term adherence, particularly early in the exercise journey. Similarly, early diversity in training modalities and greater engagement with strength-based exercise were associated with sustained participation, potentially reflecting greater skill development, or alignment with users' training preferences. The observed variation in adherence patterns by experience level further suggests that onboarding strategies tailored to users' backgrounds may be beneficial, recognizing that meaningful correlates of exercise adherence appear to evolve as individuals progress in their training. Recent advances in AI methods that integrate complex behavioral data with large language models [e.g., MotionTeller, Zhang, Pillai (44); HEALTHGURU, Wang, Griffith (45)] suggest practical ways to dynamically tailor exercise recommendations and engagement support based on users' real-world activity patterns. In particular, adaptive systems that prioritize early-frequency scaffolding, emphasize strength-oriented starter plans, and adjust exercise progressions based on users' emerging engagement patterns may help translate early consistency into durable exercise habits. Future work will be critical to evaluate whether such personalized approaches can indeed foster long-term adherence.

## 5 Conclusion

In summary, this study provides converging evidence that early consistency, structured engagement, and contextual factors are strongly associated with sustained app-recorded exercise adherence in a large cohort of mobile fitness app users. While demographic and experiential characteristics shape adherence trajectories, they were not the main determinants of dropout outcomes. By contrast, regular training patterns established during the earliest stages of app engagement emerged as a particularly robust correlate of sustained app-recorded exercise participation, thus suggesting that the initial weeks following exercise adoption represent a particularly important window for behavioral support. By leveraging large-scale, real-world behavioral data, these findings advance our understanding of how individuals transition from exercise initiation to longer-term engagement and highlight behavioral patterns that may inform future intervention designs aimed at supporting app-recorded exercise engagement and, potentially, long-term exercise habits at scale.

## Data Availability

The data analyzed in this study is subject to the following licenses/restrictions: Data was provided by Fitbod Inc. based on a data sharing agreement with the research team. Anyone wanting to access the dataset used for this study should reach out to Fitbod Inc. directly. Requests to access these datasets should be directed to https://fitbod.me/.
