# Supplementary material for: Predictors of long-term resistance exercise adherence: evidence from a large cohort of mobile app users of various experience levels
Source: Front Sports Act Living. 2026 Aug 10;8:1855668. doi: 10.3389/fspor.2026.1855668 (PMC13500638; doi:10.3389/fspor.2026.1855668)
Supplement: Supplementary file 1 [file Supplementaryfile1.docx]

**Predictors of long-term exercise adherence among beginners: Evidence from a large cohort of mobile app users**

Federica Conti^1^, Thiago Marzagão^2^, Andrew J. Galpin^1^, Brad J. Schoefield^3^

^1^Human Performance Center, Parker University, Dallas, TX, USA

^2^Fitbod Inc., San Francisco, CA, USA

^3^ Department of Exercise Science and Recreation, Lehman College, City University of New York, NY, USA

*Correspondence to Dr Federica Conti, 2540 Walnut Hill Lane, Dallas, TX 75229

**Keywords:** Exercise adherence; Physical activity; Digital health; Behavior change.

**Contents:**

**S1. Supplementary Figure 1:** Flow chart of data cleaning and preprocessing

**S2. Supplementary Table 1:** Number of beginner users at risk of dropout over the course of follow-up, stratified by sex and age

**S3.** **Cox model redundancy and collinearity analyses**

**S4. Supplementary Table 3:** Cox regression model for dropout risk in the beginner cohort

**S5. Sensitivity analyses of adherence definition**

**S6. Sensitivity analysis restricted to app-generated workouts**

**S7. Supplementary Table 11:** Sensitivity analyses of time-varying associations between early engagement behaviors and loss of adherence

**S8. Supplementary Table 12.** Time-varying Cox proportional hazards model of dropout including the frequency × workout direction interaction

**S9. Effect of experience level on long-term adherence**

**S10. Model adjustment to the proportion of externally imported workouts**

**S1. Supplementary Figure 1.** Flow chart of data cleaning and preprocessing

**S2. Supplementary Table 1:** Number of beginner users at risk of dropout over the course of follow-up, stratified by sex and age

| **Week** | **11** | **16** | **21** | **31** | **41** | **51** |
| --- | --- | --- | --- | --- | --- | --- |
| **SEX** | | | | | |  |
| **Females** | 38027 (100%) | 24806 (65.2%) | 16119 (42.4%) | 8384 (22.0%) | 4970 (13.1%) | 3253 (8.6%) |
| **Males** | 62682 (100%) | 43153 (68.8%) | 29684 (47.4%) | 16897 (27%) | 10876 (17.5%) | 7644 (12,2%) |
| **AGE GROUP** | | | | | |  |
| **18-30** | 42314 (100%) | 27302 (64.5%) | 17598 (41.6%) | 9058 (21.4%) | 5607 (13.3%) | 3788 (9.0%) |
| **31-40** | 34043 (100%) | 23278 (68.4%) | 15848 (46.6%) | 8859 (26.0%) | 5578 (16.4%) | 3803 (11.2%) |
| **41-50** | 17168 (100%) | 12194 (71.0%) | 8617 (50.2%) | 5104 (29.7%) | 4115 (24.0%) | 2277 (13.3%) |
| **51+** | 7184 (100%) | 5185 (72.2%) | 3740 (52.1%) | 2260 (31.5%) | 1820 (25.3%) | 1029 (14.3%) |

Values shown are counts at each designated timepoint, with corresponding percentages of remaining users relative to full subsamples presented in parentheses.

**S3. Cox model redundancy and collinearity analyses**

Prior to fitting the multivariable Cox regression models, candidate predictors were evaluated for redundancy and collinearity to improve model stability and interpretability. Pairwise Pearson correlation coefficients were calculated among all continuous variables to identify measures capturing overlapping constructs (cf. Supplementary Figure 2). Measures of early training frequency that were highly correlated (e.g., number of workouts and number of days with at least one workout) were consolidated, prioritising indicators of training consistency. Similarly, measures of training volume derived from all logged activity and from app-recorded workouts showed substantial correlation; to reduce redundancy and measurement heterogeneity, app-recorded workout duration was retained as a representative measure of structured exercise exposure.

Exercise composition variables expressed as percentages were also examined for mathematical dependence. Given the constrained nature of these variables, a single indicator reflecting the proportion of strength-based exercises was retained, while the remaining composition measures were excluded to avoid collinearity.

After variable consolidation, collinearity in the reduced model was formally assessed using variance inflation factors (VIFs). All VIF values were found to be below 2, indicating negligible multicollinearity (cf. Supplementary Table 2). The final Cox model for the beginner cohort therefore included a parsimonious set of behavioral, demographic, and contextual predictors selected on the basis of both empirical relationships and theoretical relevance.

**Supplementary Figure 2.** Pairwise correlations among continuous demographic and early training variables.

Values shown represent Pearson correlation coefficients between continuous predictors. Variables with conceptual overlap were consolidated prior to multivariable modelling to reduce collinearity.

**Supplementary Table 2.** Variance Inflation Factors for candidate predictors from the reduced Cox model in the beginner cohort.

| **Predictor** | **VIF** |
| --- | --- |
| Active days (first 28 days) | 1.10 |
| App workout time (first 28 days) | 1.24 |
| Strength exercises (%) | 1.41 |
| Distinct equipment types | 1.27 |
| App subscription | 1.11 |
| Sex | 1.02 |
| Age | 1.02 |

**S4. Supplementary Table 3:** Cox regression model for dropout risk in the beginner cohort

| **Predictor** | **HR (Week 11)** | **HR (Week 26)** | **HR (Week 40)** | **LR χ² (df)** | ***p*-value** |
| --- | --- | --- | --- | --- | --- |
| Active days | 0.73 [0.72–0.74] | 0.87 [0.86–0.88] | 0.91 [0.90–0.92] | 3050.35 (1) | <0.0001 |
| Active days × log(time) | — | — | — | 295.44 (1) | <0.0001 |
| Age (spline, df = 3) | — | — | — | 649.46 (3) | <0.0001 |

Hazard ratios and 95% confidence intervals estimated using a Cox proportional hazards model. Hazard ratios represent the effect per 1 standard deviation increase and are shown at specific time points to reflect time-varying effects throughout the observation period. Age was modelled using restricted cubic splines (3 degrees of freedom), sex and subscription status were stratified. Abbreviations: LR = Likelihood Ratio, df = degrees of freedom; HR = Hazard Ratio, SD = Standard Deviation.

**S5. Sensitivity analyses of adherence definition**

To assess the robustness of the primary findings to the threshold used to define long-term exercise adherence, we considered two alternative operationalization using stricter criteria. In the first case, users were required to complete at least one workout in every week of the observation period, with no missed weeks permitted. Accordingly, loss of adherence was defined as the first week in which a user failed to meet this criterion. In the second case, we increased the minimum weekly workout frequency, meaning that users were required to complete at least two workouts per week throughout the observation period, allowing up to six exceptions to account for likely real-life situations preventing exercise participation such as holidays, sick days, and other unforeseeable circumstances. In both scenarios, follow-up began on day 29 following the first recorded workout and continued through day 365.

Sensitivity analyses were conducted within the beginner cohort using the same Cox proportional hazards modeling framework as in the primary analyses. Models included time-varying effects of workout frequency, flexible spline-based modeling of age, and adjustment for sex and subscription status. Two interaction analyses were re-estimated under the stricter adherence definition: (i) the age × sex interaction and (ii) the interaction between workout frequency and app-generated workout duration, including their time-varying components. Model estimates were compared qualitatively with the primary results to assess consistency in direction, relative magnitude, and temporal patterns of association.

*No allowance for missed weeks*

When no missed weeks were allowed, adherence was found to declined steadily across time, with the steepest decrease occurring between weeks 5 and 12 (cf. Supplementary Figure 3). The median time to dropout was 9 weeks, indicating that half of all beginners ceased meeting the stricter adherence criteria by this point. Interestingly, the Kaplan-Meier survival curve was found to flatten at week 25 and, through to week 52, the estimated probability of remaining adherent was 9.87%, corresponding to approximately 9,948 beginner users.

Consistent with previous findings, the Cox regression model indicated workout frequency during the initial 28-day period was strongly associated with subsequent loss of adherence (likelihood ratio *χ*² = 5809.17, *df* = 1, *p* < 0.001). This association varied significantly over time (interaction with log(time): *χ*² = 820.84, *df* = 1, *p* < 0.001), indicating attenuation of the protective effect as follow-up progressed (cf. Supplementary Table 4). Specifically, each standard deviation increase in the number of active days was associated with a 20% lower hazard of dropout at week 11 (HR = 0.80, 95% CI: 0.793, 0.80). This association weakened but remained substantial at week 26 (HR = 0.89, 95% CI: 0.88, 0.90) and week 40 (HR = 0.94, 95% CI: 0.92, 0.95). Age was significantly associated with adherence in a non-linear fashion (*χ*² = 496.59, *df* = 3, *p* < 0.001) and was modeled using restricted cubic splines. Sex and subscription status were included as stratification factors.

**Supplementary Figure 3.** Kaplan-Meier survival curve for the beginner cohort

Estimated proportion of users who remained adherent during the observation period under the stricter adherence definition. The Kaplan–Meier curve is displayed from week 5 onward, reflecting the earliest time point at which users could meet the study definition of non-adherence, given the requirement of at least one workout per week during the initial 28-day onboarding period. Adherence declined steadily over time, with a median time to dropout of 9 weeks and an estimated 9.87% of users remaining adherent at week 52.

**Supplementary Table 4.** Cox regression model for dropout risk in the beginner cohort under the alternative adherence definition

| **Predictor** | **HR (Week 11)** | **HR (Week 26)** | **HR (Week 40)** | **LR χ² (df)** | ***p*-value** |
| --- | --- | --- | --- | --- | --- |
| Active days | 0.80 [0.79–0.80] | 0.89 [0.88–0.90] | 0.94 [0.92–0.95] | 5809.17 (1) | <0.0001 |
| Active days x log(time) | — | — | — | 820.84 (1) | <0.0001 |
| Age (spline, df = 3) | — | — | — | 496.59 (3) | <0.0001 |

Hazard ratios and 95% confidence intervals estimated using a Cox proportional hazards model. Hazard ratios represent the effect per 1 standard deviation increase and are shown at specific time points to reflect time-varying effects throughout the observation period. Age was modeled using restricted cubic splines (3 degrees of freedom), sex and subscription status were stratified. Abbreviations: LR = Likelihood Ratio, df = degrees of freedom; HR = Hazard Ratio, SD = Standard Deviation.

Under the stricter adherence definition, the significant age × sex interaction previously observed did not persisted (*χ*²(3) = 6.09, *p* = 0.11). Supplementary Figure 4 displays model-based predicted hazard ratios for age, evaluated at a representative follow-up time (week 26) and expressed relative to the cohort median age (33 years), separately for female and male users. Relative to age 33, dropout risk at age 25 was higher for both females (HR = 1.07, 95% CI: 1.05–1.08) and males (HR = 1.08, 95% CI: 1.07–1.09), whereas dropout risk at age 55 was lower (females: HR = 0.89, 95% CI: 0.86–0.92; males: HR = 0.87, 95% CI: 0.85–0.89, cf. Supplementary Table 5). Younger users exhibited higher dropout risk relative to the cohort median age regardless of sex, with risk declining across early and middle adulthood. Replicating previous findings, among women this decline was approximately monotonic across the entire lifespan, whereas among men the association reached a minimum in late middle age (58.3 years, HR = 0.87) and increased modestly at older ages.

**Supplementary Figure 4.** Age–dropout associations by sex under the stricter adherence definition

Predicted hazard ratios for dropout as a function of age, shown separately for female and male users, derived from sex-specific Cox proportional hazards models. Hazard ratios are expressed relative to the cohort median age and are evaluated at a representative follow-up time (week 26). Models were adjusted for early workout frequency with a time-varying effect and stratified by subscription status.

**Supplementary Table 5.** Age-specific hazard ratios by sex under the alternative adherence definition

|  | **Hazard Ratios [95% CI]** | | |
| --- | --- | --- | --- |
| **Sex** | **Age 25** | **Age 40** | **Age 55** |
| Female | 1.07 [1.05-1.08] | 0.96 [0.94-0.97] | 0.89 [0.86-0.92] |
| Male | 1.08 [1.07-1.09] | 0.93 [0.92-0.95] | 0.87 [0.85-0.89] |

Model-based hazard ratios (HRs) and 95% confidence intervals for dropout risk at ages 25, 40, and 55 years, relative to the cohort median age (33 years), shown separately for females and males. Estimates are derived from spline-based Cox proportional hazards models including an age × sex interaction term and evaluated at week 26. Models were fitted within the beginner cohort using a stricter adherence definition (no missed weeks permitted), adjusted for workout frequency, and stratified by subscription status.

A likelihood ratio test comparing models with and without the workout frequency × duration interaction indicated strong evidence of effect modification (*χ*²(1) = 91.25, *p* < 0.001), with further model comparisons supporting time-varying moderation of this interaction over follow-up (*χ*²(1) = 18.83, *p* < 0.001). In the fully adjusted Cox model, workout frequency showed a strong protective association early in follow-up (i.e. week 11; β = −0.43, HR = 0.65, *p* < 0.001), an effect found to attenuate over time (workout frequency x log(time): β = 0.10, HR = 1.11, *p* < 0.001). Importantly, the protective effect of workout frequency was stronger among users completing longer app-generated workouts, as reflected by a significant frequency × duration interaction (β = −0.06, HR = 0.94, *p* < 0.001). This duration-based moderation was also found to diminish over time (workout frequency × duration × log(time): β = 0.01, HR = 1.02, *p* < 0.001). Consistent with these effects, time-specific hazard ratios derived from the fitted model indicated that a one–standard deviation increase in workout frequency was associated with a 22% reduction in dropout risk at week 11 among users completing longer workouts, compared with a 17% reduction among users completing shorter workouts, with differences progressively reducing over time (cf. Supplementary Table 6).

**Supplementary Table 6.** Time-specific hazard ratios for dropout by workout frequency and app-generated workout duration under the alternative adherence definition

| **Duration** | **HR (Week 11)** | **HR (Week 26)** | **HR (Week 40)** |
| --- | --- | --- | --- |
| Short (-1 SD) | 0.83 [0.82-0.83] | 0.91 [0.90-0.93] | 0.95 [0.94-0.97] |
| Average | 0.80 [0.80-0.81] | 0.90 [0.89-0.91] | 0.95 [0.94-0.96] |
| Long (+1 SD) | 0.78 [0.77-0.79] | 0.89 [0.87-0.90] | 0.94 [0.92-0.97] |

Model-based hazard ratios (HRs) and 95% confidence intervals for dropout risk associated with a one–standard deviation increase in workout frequency, evaluated at weeks 11, 26, and 40, separately for short (−1 SD), average (0 SD), and long (+1 SD) app-generated workout durations. Estimates are derived from Cox proportional hazards models fitted within the beginner cohort using a stricter adherence definition (no missed weeks permitted). Models adjust for age (modeled using restricted cubic splines), sex, and are stratified by subscription status.

*Two-workout per week requirement*

When long-term exercise adherence was defined as completing at least two workout per weeks throughout the observation period, adherence was once again found to declined steadily across time, with the steepest decrease occurring between weeks 5 and 12 (cf. Supplementary Figure 5). The median time to dropout was 12 weeks, indicating that half of all beginners ceased meeting the stricter adherence criteria by this point. At week 52, the estimated probability of remaining adherent was 6.48%, corresponding to approximately 6,531 beginner users.

The Cox regression model indicated workout frequency during the initial 28-day period was strongly associated with subsequent loss of adherence (likelihood ratio *χ*² = 6276.33, *df* = 1, *p* < 0.001). This association varied significantly over time (interaction with log(time): *χ*² = 787.02, *df* = 1, *p* < 0.001), indicating attenuation of the protective effect as follow-up progressed (cf. Supplementary Table 7). Specifically, each standard deviation increase in the number of active days was associated with a 20% lower hazard of dropout at week 11 (HR = 0.80, 95% CI: 0.793, 0.80). This association weakened but remained substantial at week 26 (HR = 0.89, 95% CI: 0.88, 0.90) and week 40 (HR = 0.94, 95% CI: 0.92, 0.95). Age was significantly associated with adherence in a non-linear fashion (*χ*² = 819.05, *df* = 3, *p* < 0.001) and was modeled using restricted cubic splines. Sex and subscription status were included as stratification factors.

**Supplementary Figure 5.** Kaplan-Meier survival curve for the beginner cohort when completing at least two workout sessions per week

Estimated proportion of users who remained adherent during the observation period by completing at least two workout sessions per week. The Kaplan–Meier curve is displayed from week 5 onward, reflecting the earliest time point at which users could meet the study definition of non-adherence, given the requirement of at least one workout per week during the initial 28-day onboarding period. Adherence declined steadily over time, with a median time to dropout of 12 weeks and an estimated 6.48% of users remaining adherent at week 52.

**Supplementary Table 7.** Cox regression model for dropout risk in the beginner cohort when completing at least two workout sessions per week

| **Predictor** | **HR (Week 11)** | **HR (Week 26)** | **HR (Week 40)** | **LR χ² (df)** | ***p*-value** |
| --- | --- | --- | --- | --- | --- |
| Active days | 0.64 [0.63–0.65] | 0.84 [0.83–0.85] | 0.89 [0.88–0.91] | 6276.33 (1) | <0.0001 |
| Active days x log(time) | — | — | — | 787.02 (1) | <0.0001 |
| Age (spline, df = 3) | — | — | — | 819.05 (3) | <0.0001 |

Hazard ratios and 95% confidence intervals estimated using a Cox proportional hazards model. Hazard ratios represent the effect per 1 standard deviation increase and are shown at specific time points to reflect time-varying effects throughout the observation period. Age was modeled using restricted cubic splines (3 degrees of freedom), sex and subscription status were stratified. Abbreviations: LR = Likelihood Ratio, df = degrees of freedom; HR = Hazard Ratio, SD = Standard Deviation.

The significant age × sex interaction observed in the original model persisted (*χ*²(3) = 11.83, *p* = 0.008). Supplementary Figure 6 displays model-based predicted hazard ratios for age, evaluated at a representative follow-up time (week 26) and expressed relative to the cohort median age (33 years), separately for female and male users. Relative to age 33, dropout risk at age 25 was higher for both females (HR = 1.09, 95% CI: 1.07–1.11) and males (HR = 1.12, 95% CI: 1.11–1.13), whereas dropout risk at age 55 was lower (females: HR = 0.88, 95% CI: 0.85–0.91; males: HR = 0.85, 95% CI: 0.83–0.88, cf. Supplementary Table 8). Younger users exhibited higher dropout risk relative to the cohort median age regardless of sex, with risk declining across early and middle adulthood. Replicating previous findings, among women this decline was approximately monotonic across the entire lifespan, whereas among men the association reached a minimum in late middle age (66.89 years, HR = 0.84) and increased modestly at older ages.

**Supplementary Figure 6.** Age–dropout associations by sex when completing at least two workout sessions per week

Predicted hazard ratios for dropout as a function of age, shown separately for female and male users, derived from sex-specific Cox proportional hazards models. Hazard ratios are expressed relative to the cohort median age and are evaluated at a representative follow-up time (week 26). Models were adjusted for early workout frequency with a time-varying effect and stratified by subscription status.

**Supplementary Table 8.** Age-specific hazard ratios by sex when completing at least two workout sessions per week

|  | **Hazard Ratios [95% CI]** | | |
| --- | --- | --- | --- |
| **Sex** | **Age 25** | **Age 40** | **Age 55** |
| Female | 1.09 [1.07-1.11] | 0.95 [0.94-0.97] | 0.88 [0.85-0.91] |
| Male | 1.12 [1.11-1.13] | 0.93 [0.92-0.94] | 0.85 [0.83-0.88] |

Model-based hazard ratios (HRs) and 95% confidence intervals for dropout risk at ages 25, 40, and 55 years, relative to the cohort median age (33 years), shown separately for females and males. Estimates are derived from spline-based Cox proportional hazards models including an age × sex interaction term and evaluated at week 26. Models were fitted within the beginner cohort, requiring the completion of at least two workout sessions per week, and were adjusted for workout frequency, and stratified by subscription status.

A likelihood ratio test comparing models with and without the workout frequency × duration interaction indicated strong evidence of effect modification (*χ*²(1) = 69.37, *p* < 0.001), with further model comparisons supporting time-varying moderation of this interaction over follow-up (*χ*²(1) = 29.34, *p* < 0.001). In the fully adjusted Cox model, workout frequency showed a strong protective association early in follow-up (i.e. week 11; β = −0.45, HR = 0.64, *p* < 0.001), an effect found to attenuate over time (workout frequency x log(time): β = 0.10, HR = 1.11, *p* < 0.001). Once again, the protective effect of workout frequency was stronger among users completing longer app-generated workouts, as reflected by a significant frequency × duration interaction (β = −0.06, HR = 0.94, *p* < 0.001). This duration-based moderation was also found to diminish over time (workout frequency × duration × log(time): β = 0.02, HR = 1.02, *p* < 0.001). Consistent with these effects, time-specific hazard ratios derived from the fitted model indicated that a one–standard deviation increase in workout frequency was associated with a 40% reduction in dropout risk at week 11 among users completing longer workouts, compared with a 32% reduction among users completing shorter workouts (cf. Supplementary Table 9).

**Supplementary Table 9.** Time-specific hazard ratios for dropout by workout frequency and app-generated workout duration when completing at least two workout sessions per week

| **Duration** | **HR (Week 11)** | **HR (Week 26)** | **HR (Week 40)** |
| --- | --- | --- | --- |
| Short (-1 SD) | 0.68 [0.66-0.69] | 0.86 [0.85-0.87] | 0.90 [0.89-0.92] |
| Average | 0.64 [0.63-0.65] | 0.85 [0.84-0.85] | 0.90 [0.89-0.92] |
| Long (+1 SD) | 0.60 [0.58-0.61] | 0.84 [0.82-0.85] | 0.90 [0.89-0.92] |

Model-based hazard ratios (HRs) and 95% confidence intervals for dropout risk associated with a one–standard deviation increase in workout frequency, evaluated at weeks 11, 26, and 40, separately for short (−1 SD), average (0 SD), and long (+1 SD) app-generated workout durations. Estimates are derived from Cox proportional hazards models fitted within the beginner cohort requiring the completion of at least two workout sessions per week. Models adjust for age (modeled using restricted cubic splines), sex, and are stratified by subscription status.

**S6. Sensitivity analysis restricted to app-generated workouts**

To assess the robustness of the primary findings to potential measurement error arising from imported workout sessions, we repeated the main survival analysis in a subset of beginner users who completed exclusively app-generated workouts during the initial 28-day period. Results were highly consistent with the primary analysis (cf. Supplementary Table 10). Early workout frequency remained strongly associated with reduced dropout risk, with a one–standard deviation increase corresponding to a 75% lower hazard at week 11 (HR = 0.75, 95% CI: 0.73–0.77), attenuating to an 11% reduction at week 26 (HR = 0.89, 95% CI: 0.88–0.90) and an 8% reduction at week 40 (HR = 0.92, 95% CI: 0.91–0.94). The persistence of both the direction and time-varying nature of the association suggests that the main findings are not driven by the inclusion of imported workout data.

**Supplementary Table 10.** Sensitivity analysis comparing primary and app-only models in beginner users

| **Follow-up time** | **Primary model HR (95%)** | **App-only model HR (95% CI)** |
| --- | --- | --- |
| Week 11 | 0.73 [0.72–0.74] | 0.75 [0.73–0.77] |
| Week 26 | 0.87 [0.86–0.88] | 0.89 [0.88–0.90] |
| Week 40 | 0.91 [0.90-0.92] | 0.92 [0.91-0.94] |

Comparison of time-specific hazard ratios for dropout associated with early workout frequency in beginners. Estimates from the primary model (including both app-generated and imported workouts) are shown alongside estimates from a sensitivity analysis restricted to users completing exclusively app-generated workouts during the initial 28-day period. Hazard ratios correspond to a one–standard deviation increase in active training days and are evaluated at weeks 11, 26, and 40 of follow-up.

**S7. Supplementary Table 11:** Sensitivity analyses of time-varying associations between early engagement behaviors and loss of adherence

| **Predictor** | **HR (Week 11)** | **HR (Week 26)** | **HR (Week 40)** |
| --- | --- | --- | --- |
| Active days | 0.73 [0.72, 0.74] | 0.87 [0.86, 0.88] | 0.91 [0.90, 0.92] |
| App workout time | 0.83 [0.81, 0.84] | 095 [0.94, 0.96] | 0.98 [0.97, 0.99] |
| Strength exercises (%) | 0.77 [0.76, 0.78] | 0.99 [0.95, 0.97] | 1.01 [1.00, 1.02] |
| Distinct equipment types | 0.72 [0.71, 0.74] | 0.91 [0.91, 0.92] | 0.96 [0.95, 0.97] |

Hazard ratios and 95% confidence intervals estimated using a Cox proportional hazards model. Hazard ratios represent the effect per 1 standard deviation increase and are shown at specific time points to reflect time-varying effects throughout the observation period. Each engagement measure was modeled in a separate Cox proportional hazards model as the sole time-varying predictor. Age was modeled using restricted cubic splines (3 degrees of freedom), sex and subscription status were stratified. Abbreviations: HR = Hazard Ratio.

**S8. Supplementary Table 12.** Time-varying Cox proportional hazards model of dropout including the frequency × workout direction interaction

| **Predictor** | **β** | **HR** | **95% CI** | **SE** | | **z** | **p-value** |
| --- | --- | --- | --- | --- | --- | --- | --- |
| Workout frequency | −0.32 | 0.73 | [0.71–0.74] | 0.009 | | −37.14 | <0.001 |
| App workout duration | −0.06 | 0.94 | [0.93–0.94] | 0.004 | −17.52 | | <0.001 |
| Age (spline term 1) | −0.35 | 0.70 | [0.67–0.74] | 0.025 | −14.13 | | <0.001 |
| Age (spline term 2) | −0.54 | 0.58 | [0.52–0.65] | 0.059 | −9.26 | | <0.001 |
| Age (spline term 3) | −0.26 | 0.77 | [0.63–0.93] | 0.099 | −2.64 | | 0.008 |
| Male sex | −0.12 | 0.89 | [0.87–0.90] | 0.007 | −17.43 | | <0.001 |
| Workout frequency × log(time) | 0.07 | 1.07 | [1.06–1.08] | 0.004 | 18.32 | | <0.001 |
| Frequency × duration | −0.08 | 0.93 | [0.91–0.94] | 0.008 | −9.37 | | <0.001 |
| Frequency × duration × log(time) | 0.02 | 1.02 | [1.01–1.03] | 0.004 | 5.78 | | <0.001 |

Workout frequency and app-generated workout duration were z-standardized prior to modeling. Hazard ratios represent effects per 1 standard deviation increase. Age was modeled using restricted cubic splines (3 degrees of freedom). Models were estimated using a counting-process formulation with the Efron method for ties and were stratified by subscription status. Coefficients for workout frequency represent time-dependent effects; interaction terms indicate modification of the frequency effect by workout duration and follow-up time. β coefficients represent log hazard ratios estimated from the Cox proportional hazards model. Abbreviations: HR = Hazard Ratio, SE = Standard Error.

**S9. Effect of experience level on long-term adherence**

**Supplementary Figure 7.** Kaplan-Meier survival curves by self-reported experience level

Estimated proportion of users who remained adherent during the observation period, stratified by experience level. The Kaplan–Meier curve is displayed from week 11 onward, reflecting the earliest time point at which users could meet the study definition of non-adherence, given the requirement of at least one workout per week during the initial 28-day onboarding period and allowance for up to six weeks with no active days. Probability of adherence was comparatively higher across the full follow-up period in users with higher training experience. The difference in survival distributions was significant according to the log-rank test (*p* < 0.001).

**Supplementary Table 13.** Number of users at risk of dropout, stratified by self-reported experience level

| **Week** | **11** | **16** | **21** | **31** | **41** | **51** |
| --- | --- | --- | --- | --- | --- | --- |
| **Beginners** | 100709 (100%) | 67959 (67.5%) | 45803 (45.5%) | 25281 (25.1%) | 15846 (15.8%) | 10897 (10.8%) |
| **Intermediate** | 193621 (100%) | 149567 (77.2%) | 113779 (58.8%) | 73335 (37.9%) | 51213 (26.5%) | 37542 (19.4%) |
| **Advanced** | 95151 (100%) | 78448 (82.4%) | 63267 (66.5%) | 44928 (47.2%) | 33538 (35.2%) | 25819 (27.1%) |

Values shown are counts at each designated timepoint, with corresponding percentages of remaining users relative to full subsamples presented in parentheses.

**Supplementary Table 14.** Time-varying Cox proportional hazards model of dropout, stratified by experience level

| **Predictor** | **β** | **HR** | **95% CI** | **p-value** |
| --- | --- | --- | --- | --- |
| Workout frequency | −0.30 | 0.74 | [0.74–0.75] | <0.001 |
| Age (spline term 1) | −0.35 | 0.71 | [0.69–0.73] | <0.001 |
| Age (spline term 2) | −0.58 | 0.56 | [0.53–0.60] | <0.001 |
| Age (spline term 3) | −0.22 | 0.81 | [0.72–0.91] | <0.001 |
| Male sex | −0.15 | 0.86 | [0.86–0.87] | <0.001 |
| Workout frequency × log(time) | 0.05 | 1.06 | [1.05–1.06] | <0.001 |

Hazard ratios (HRs) and 95% confidence intervals are shown. Workout frequency is standardized (per 1 SD increase). Age was modeled using restricted cubic splines (3 degrees of freedom). Models are stratified by experience level (beginner, intermediate, advanced) and subscription status; hazard ratios for these variables are therefore not estimated. Time-varying effects were modeled via interaction with log-transformed follow-up time. β coefficients represent log hazard ratios estimated from the Cox proportional hazards model. Abbreviations: HR = Hazard Ratio, CI = Confidence Interval.

**Supplementary Table 15**. Age-specific hazard ratios for dropout by sex and experience level

|  | **Hazard Ratios [95% CI]** | | |
| --- | --- | --- | --- |
| **Beginners** | **Age 25** | **Age 40** | **Age 55** |
| Female | 1.09 [1.07-1.10] | 0.97 [0.95-0.98] | 0.90 [0.88-0.93] |
| Male | 1.12 [1.11-1.13] | 0.95 [0.94-0.96] | 0.88 [0.86-0.90] |
| **Intermediate** |  |  |  |
| Female | 1.09 [1.08-1.10] | 0.97 [0.96-0.98] | 0.90 [0.88-0.92] |
| Male | 1.15 [1.15-1.16] | 0.95 [0.94-0.96] | 0.89 [0.88-0.91] |
| **Advanced** |  |  |  |
| Female | 1.04 [1.01-1.07] | 0.98 [0.96-1.00] | 0.97 [0.93-1.00] |
| Male | 1.17 [1.16-1.18] | 0.96 [0.95-0.97] | 0.94 [0.92-0.97] |

Model-based hazard ratios (HRs) and 95% confidence intervals for dropout risk at ages 25, 40, and 55 years, expressed relative to the cohort median age (33 years). Estimates are shown separately for females and males within beginner, intermediate, and advanced user cohorts. HRs were derived from Cox proportional hazards models with age modeled using restricted cubic splines and an age × sex interaction term, evaluated at week 26 of follow-up. Models adjust for workout frequency and are stratified by subscription status.

**S10. Model adjustment to the proportion of externally imported workouts**

Because the dataset included both app-generated and externally imported workout sessions, an additional sensitivity analysis was conducted to assess whether differences in workout logging practices influenced the primary results. Specifically, we computed, for each user, the proportion of workouts during the initial 28-day period that were externally imported rather than app-generated and included this measure as an additional covariate in the Cox proportional hazards model.

In this adjusted model, early workout frequency remained a strong predictor of lower dropout risk, and its time-varying attenuation over follow-up was preserved (HR = 0.80, 95% CI: 0.78–0.82, cf. Supplementary Table 16). The magnitude and direction of hazard ratios were similar to those observed in the primary model.

The proportion of imported workouts was independently associated with higher dropout risk (HR = 1.10, 95% CI: 1.06–1.14), suggesting that users who relied more heavily on imported sessions during onboarding were less likely to remain adherent over time. Nonetheless, the inclusion of this variable did not materially alter the estimated effects of early workout frequency or age.

**Supplementary Table 16.** Cox proportional hazards model of dropout risk adjusted for the proportion of imported workouts in the beginner cohort

| **Predictor** | **β** | **HR** | **95% CI** | **p-value** |
| --- | --- | --- | --- | --- |
| Workout frequency | −0.22 | 0.80 | [0.78–0.82] | <0.001 |
| Workout frequency × log(time) | 0.03 | 1.03 | [1.02-1.04] | <0.001 |
| Proportion of imported workouts | 0.10 | 1.10 | [1.06-1.14] | <0.001 |
| Age (spline term 1) | −0.33 | 0.72 | [0.67–0.78] | <0.001 |
| Age (spline term 2) | −0.26 | 0.77 | [0.65–0.92] | <0.001 |
| Age (spline term 3) | −0.05 | 0.95 | [0.71–1.26] | 0.7 |

Hazard ratios (HRs) and 95% confidence intervals are shown. Workout frequency is standardized (per 1 SD increase). Age was modeled using restricted cubic splines (3 degrees of freedom). Models are stratified by sex and subscription status; hazard ratios for these variables are therefore not estimated. Workout frequency and duration variables are standardised (z-scored). Time-varying effects were modeled via interaction with log-transformed follow-up time. β coefficients represent log hazard ratios estimated from the Cox proportional hazards model. Abbreviations: HR = Hazard Ratio, CI = Confidence Interval.
